# Involvement of HDAC1 and HDAC3 in the Pathology of Polyglutamine Disorders: Therapeutic Implications for Selective HDAC1/HDAC3 Inhibitors

**DOI:** 10.3390/ph7060634

**Published:** 2014-05-26

**Authors:** Elizabeth A. Thomas

**Affiliations:** Department of Molecular and Cellular Neuroscience, The Scripps Research Institute, SP2030 10550 N. Torrey Pines Rd, La Jolla, CA 92037, USA; E-Mail: bthomas@scripps.edu; Tel.: +1-858-784-2317; Fax: +1-858-784-2212

**Keywords:** histone deacetylase (HDAC), HDAC1, HDAC3, subtype, selective, neurodegenerative, polyglutamine, histone, chromatin, mechanism

## Abstract

Histone deacetylases (HDACs) enzymes, which affect the acetylation status of histones and other important cellular proteins, have been recognized as potentially useful therapeutic targets for a broad range of human disorders. Emerging studies have demonstrated that different types of HDAC inhibitors show beneficial effects in various experimental models of neurological disorders. HDAC enzymes comprise a large family of proteins, with18 HDAC enzymes currently identified in humans. Hence, an important question for HDAC inhibitor therapeutics is which HDAC enzyme(s) is/are important for the amelioration of disease phenotypes, as it has become clear that individual HDAC enzymes play different biological roles in the brain. This review will discuss evidence supporting the involvement of HDAC1 and HDAC3 in polyglutamine disorders, including Huntington’s disease, and the use of HDAC1- and HDAC3-selective HDAC inhibitors as therapeutic intervention for these disorders. Further, while HDAC inhibitors are known alter chromatin structure resulting in changes in gene transcription, understanding the exact mechanisms responsible for the preclinical efficacy of these compounds remains a challenge. The potential chromatin-related and non-chromatin-related mechanisms of action of selective HDAC inhibitors will also be discussed.

## 1. Introduction

A growing body of literature suggests that epigenetic dysregulation is a key pathogenic feature of many polyglutamine disorders [1,2,3]. Given the prominent role of histone modifying enzymes to alter gene expression, the use of histone deacetylase (HDAC) inhibitors as a potential therapeutic approach for polyglutamine has gained considerable attention in recent years. Initial preclinical studies testing HDAC inhibitors in mouse models of polyglutamine disorders were carried out using broad-spectrum HDAC inhibitors; however, the clinical use of these broadly-acting compounds for neurodegenerative disorders is limited by their known toxicity, hence, it is becoming clear that inhibitors with subtype-selectivity may prove to be more beneficial for targeting neurological disease symptoms and minimizing harmful side effects [4]. An essential issue for HDAC inhibitor therapeutics, is the knowledge of which HDAC enzyme(s) is/are important for the clinical efficacy of these compounds for their target disease. The HDACs comprise a large family of proteins, with18 HDAC enzymes having been identified in humans [5], and studies over the past five years have demonstrated that individual HDAC enzymes play vastly different roles in the CNS. Several HDAC family members, including HDAC1 and HDAC3, have been implicated in neurotoxicity and in neuropathological mechanisms related to polyglutamine disease states (see below). Further, recent findings have demonstrated that class I-specific and HDAC1/HDAC3-selective HDAC inhibitors are effective in suppressing pathogenic symptoms in various model systems. This review will summarize the involvement of HDACs 1 and 3 in HD, and other polyglutamine disorders, providing a basis for the use of selective HDAC inhibitors targeting these subtypes for therapeutic purposes.

## 2. Gene Expression Regulation: Chromatin, HATs and HDACs

Gene expression is essentially dependent on factors that alter chromatin structure. The basic unit of chromatin is the nucleosome, which consists of 147 base pairs of DNA wrapped 1.6 times around an octamer of core histone proteins H2A, H2B, H3 and H4 [6]. The amino-terminal tails of these core histones contain amino acid residues that are sites for acetylation, methylation, phosphorylation and ubiquitination; these posttranslational modifications alter histone interactions with DNA and nuclear proteins, resulting in changes in gene transcription [7]. The “histone code” refers to the specific patterns of modified histones, which correspond to various states of chromatin and to the activation or repression of distinct sets of genes [8]. One of the best-studied histone post-translational modifications is acetylation, the transfer of an acetyl group from acetyl coenzyme A to the -lysine side chain in the acceptor histone. Histone acetylation and deacetylation of histones are modulated by the actions of two opposing enzymes, histone acetyltransferases (HATs) and HDACs [7,8]. In general, increases in HAT activity promote acetylation of histone proteins leading to increased gene transcription by creating a more open conformation of chromatin. In contrast, HDAC activity involves removing the acetyl group from histones, which results in a decrease in the space between the nucleosome and the DNA that is wrapped around it, resulting in condensation of chromatin structure and ensuing repression of gene expression. However, the precise mechanisms of transcriptional regulation are likely to be more complex and involve many other chromatin-related proteins.

HATs makes up a diverse family of proteins, including Gcn5-related N-acetyltransferase superfamily members, MYST proteins, global coactivators p300 and CREB-binding protein, nuclear receptor coactivators, TATA-binding protein-associated factor TAF(II)250 and its homologs and subunits of RNA polymerase III general factor TFIIIC [9]. A review of this family of enzymes has been summarized elsewhere [10]. In contrast, HDAC enzymes comprise a more related family of proteins with structural similarities.

## 3. HDAC Family of Proteins

HDAC proteins constitute an ancient enzyme family, conserved in evolution from yeast to plants and animals [11]. HDAC-like proteins are found as well in Eubacteria and Archaebacteria [12]. In mammals, the HDAC enzymes comprise a large family of proteins, with 18 HDAC subtypes identified in humans [5]. These enzymes have been divided into distinct groups. Class I HDACs consists of HDACs 1, 2, 3 and 8, while class II HDACs are divided into two groups: class IIa, consisting of HDACs 4, 5, 7 and 9, and class IIb, consisting of HDACs 6 and 10 [5]. Class II enzymes share significant sequence and structural homology and, like class I HDACs, require zinc for catalytic activity. Members of a third class of HDACs, called the “sirtuins”, are structurally unrelated from classes I and II and require NAD^+^ for their enzymatic activity [13]. Class IV is represented by a single member, HDAC11 [14], and while sharing similar characteristics to HDACs in classes I and II, HDAC 11 is thought to have a distinct physiological role [14,15]. HDACs exist in large multiprotein complexes, and much evidence suggests that most, if not all, HDAC enzymes require interaction with other HDACs or proteins for optimal enzymatic activity [16,17]. HDACs lack a DNA-binding motif and one function of HDAC-interacting proteins is recruitment to their chromatin targets [18]. HDAC-containing repressor complexes consist of a multitude of components and it is important to note that class I HDAC-containing complexes may have different subunits in different cell types, at specific developmental stages or depending on the purification methods. Different HDAC enzymes have been implicated in neurodegenerative disorders [19]. In HD, altered expression of HDACs 2, 4 5 and 6 have been reported in HD model systems [20,21,22,23] and specific reduction of HDAC4 and HDAC6 has been shown to improve HD disease phenotypes [24,25]. However, for the purpose of this review, we will further describe only HDACs 1 and 3, and discuss the growing evidence for their roles in polyglutamine disorders.

### 3.1. HDAC1

HDAC1 is perhaps the most widely studied of all the HDAC enzymes. HDAC1 exhibits a high degree of homology to HDAC2 (85% of global sequence identity) and has a high degree of functional overlap with HDAC2 for many biological processes [11,26]. However, it has become evident from knockout studies that HDAC1 and HDAC2 also have distinct and non-redundant biological functions [27]. For example, germ-line deletion of *Hdac1* results in early embryonic lethality by embryonic day 9.5, whereas mice lacking *Hdac2* survive embryogenesis and either die shortly after birth [28] or survive to adulthood in others [29,30], depending on the model. Although the expression of HDAC2 and HDAC3 is increased in *Hdac1*-deficient cells, they cannot compensate for loss of the enzyme, suggesting unique biological functions for HDAC1. Like all class I HDACs, HDAC1 is primarily localized to the nucleus, however, studies have also shown cytoplasmic expression of HDAC1 [31]. HDAC1 is expressed within the brain, albeit at lower levels than other HDACs, such as HDAC3 and HDAC11 [32,33]. It is most abundantly expressed in the cerebellum, followed by amygdala and hippocampus [32,33]. HDAC1 is expressed primarily in neurons but also in glial cells, including astrocytes [32,34] and oligodendrocytes, where it is required for formation and differentiation [35].

**Figure 1:**
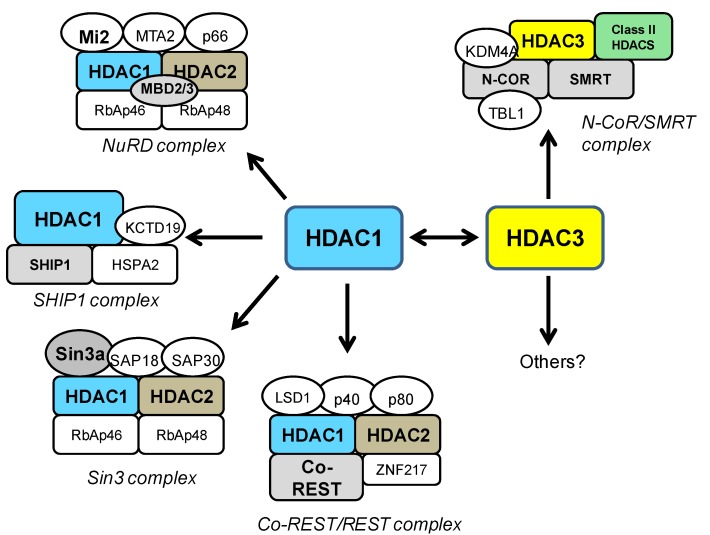
Schematic depiction of HDAC1- and HDAC3-containing co-repressor complexes. HDAC1 and 2 form a heterodimer and constitute the catalytic core of the Sin3, NuRD, and CoREST complexes [18], while the recently described SHIP1 complex contains only HDAC1 [36]. HDAC3 is a major component of the NCoR/SMRT complex [18], where it can interact with class II HDACs. Class I HDAC-containing complexes may have different subunits in different cell types, at specific developmental stages or depending on the purification methods [18]. Some of the important pathways for HDAC3 have been identified, but other chromatin targets of HDAC3 likely remain to be identified. The Sin3 complex: the transcriptional co-repressor Sin3, Sin3 associated proteins (SAP18 and 30), Rb associated proteins (RbAp46 and 48), HDAC1 and HDAC2. The NuRD complex: RbAP46 and 48, chromatin remodeler Mi2, methyl CpG binding domain proteins (MBD2/MBD3), the transcriptional repressor p66, Metastasis-associated gene family, member 2 (MTA2), HDAC1 and HDAC2. The CoREST/REST complex: co-repressor CoREST, the lysine-specific demethylase (LSD1), the Kruppel-like zinc-finger protein (ZNF217), p40, p80, HDAC1 and HDAC2. The SHIP1 complex: Heat shock 70kDa protein 2 (HSPA2), Potassium channel tetramerisation domain containing 19 (KCTD19), spermatogenic cell HDAC-interacting protein 1 (SHIP1), HDAC1. The NCoR/SMRT complex: Nuclear receptor CoRepressor (NCOR), Silencing Mediator for Retinoid and Thyroid receptor (SMRT), transducin β-like 1 (TBL1), lysine-specific demethylase (KDM4A), HDAC3.

HDAC1 can interact with several other HDAC proteins, but most notably, it functions in combination with HDAC2 in several repressor complexes. HDAC1 and HDAC2 form a heterodimer which constitutes the catalytic core of the Sin3a, NuRD, and REST/CoREST complexes (Figure 1). The Sin3 and the NuRD complexes are broadly-acting modulators of gene transcription [26,27] and bind a spectrum of different cofactors such as SAP proteins, Mi2, MTA2, and MBD proteins [18,25,28,29]. On the other hand, the REST/CoREST complex has more specific functions in the transcriptional repression of neural genes [30,31]. More recently, another HDAC1-containing complex has been characterized, the SHIP1 (spermatogenic cell HDAC-interacting protein 1) complex [36]. SHIP1 is a DNA-remodeling protein involved in chromatin dynamics during spermatogenesis, which interacts specifically with HDAC1, but not HDAC2 [36] (Figure 1).

### 3.2. HDAC3

HDAC3 is the third HDAC identified in mammals by sequence homology with previously identified HDAC1 and HDAC2 [37]. While its primary localization is in the nucleus, HDAC3 can shuttle between the nucleus and cytoplasm [38]. Germ-line deletion of *Hdac3* is lethal, and embryos die before day 9.5 [39], indicating a requirement for early embryonic development. Conditional knockout of *Hdac3* in mouse embryonic fibroblast cells have been carried out, revealing that the transcription of a variety of genes involved in metabolism, cell cycle, apoptosis, development, and signal transduction are regulated by HDAC3 and could contribute to the observed development lethality [39]. HDAC3 is found in many tissues throughout the body, including the brain [40]. It is the most highly expressed class I HDAC in the brain with greatest expression in the hippocampus, cortex, and cerebellum, but also shows high levels in striatum, amygdala and hypothalamus [32,33]. HDAC3 is predominantly expressed in neurons, but studies have also shown expression in glial cells, including astrocytes and oligodendrocytes [32,41,42].

HDAC3 shares structural and functional features with other class I HDACs, but it exists in multi-subunit complexes that are different from other known HDAC complexes. HDAC3 is most commonly found in transcription co-repressor complexes containing the nuclear receptor corepressor (NCoR) and silencing mediator for retinoid and thyroid receptors (SMRT), which regulates transcriptional repression of a wide range of genes (Figure 1) [43]. While HDAC3 is the primary HDAC enzyme in NCoR/SMRT complexes, other HDACs can be recruited in a transcription factor-specific or context-specific manner [44,45,46]. Several other HDAC proteins have been shown to interact with HDAC3. In particular, class II HDACs (4, 5, 7, and 10) have been shown to interact with HDAC3 in NCoR/SMRT complexes [44,45,46]. Specifically, HDAC4 was found to co-immunoprecipitate with HDAC3 via its C-terminal domain and disruption of this interaction resulted in loss of observed HDAC activity. Importantly, it has been indicated that the HDAC domains of HDAC4 and HDAC5 do not possess intrinsic enzymatic activity as isolated polypeptides but are associated with HDAC activity only by interacting with HDAC3, via the transcriptional corepressor NCoR/SMRT [44,45,46]. Interestingly, recent work has suggested that class IIa HDACs are not HDACs at all, but rather acetyl-lysine binding proteins, that recruit HDAC3 to active chromatin in order to alter gene transcription [47]. Co-immunoprecipitation studies have also demonstrated a direct interaction between HDAC3 and HDAC1 both when co-expressed in HEK293 cells, as well as in cultured neurons [48]. In that study, the HDAC1-HDAC3 interaction was shown to promotes neurotoxic effects (see below) [48]. While some of the important pathways for HDAC3 have been identified, many other chromatin targets or interacting proteins of HDAC3 likely remain to be identified in different cellular settings.

## 4. HDAC1 and HDAC3 in General Neurotoxicity

Neurotoxic effects for both HDAC1 and HDAC3 have been demonstrated in different model systems. Cell transfection studies demonstrated that elevating HDAC3 expression promotes the death of rat cerebellar granule and cortical neurons, but had no effect on the viability of primary kidney fibroblasts and HeLa cells, indicating that HDAC3-induced toxicity is cell-selective and that neuronal cells are most vulnerable [49]. Accordingly, shRNA-mediated suppression of HDAC3 expression protected against potassium deprivation-induced neuronal death [49], further implicating a specific role for HDAC3.

HDAC1 over-expression was also demonstrate to induce death of cerebellar granule neurons and cortical neurons in culture [48]. Interestingly, the neurotoxic effects of HDAC1 required interaction and cooperation with HDAC3. Genetic knock-down of HDAC3 was found to suppress HDAC1-induced neurotoxicity, and correspondingly, genetic knock-down of HDAC1 was found to reduce HDAC3 neurotoxicity. The HDAC1-HDAC3 interaction was greatly elevated under conditions of neurodegeneration both *in vitro* and *in vivo*, suggesting importance to disease pathology. Mechanistic studies implicated that the effects of both HDAC1 and HDAC3 were dependent on GSK3β activity and could be inhibited by IGF-1 treatment or activation of PI3K-Akt signaling [48,49]. Other studies have reported a cytotoxic role for HDAC1 that is independent of its nuclear function and occurs upon export to the cytoplasm [50]. This toxic effect of HDAC1 is linked to a mechanism impairing mitochondrial transport in damaged neurons [50].

HDAC1 has also been associated with neuroprotective effects. Previous studies have shown that Histone deacetylase-related protein (HDRP), an alternatively spliced and truncated form of HDAC9 that lacks a C-terminal catalytic domain, protects neurons from death [51]. The neuroprotective effect of HDRP requires deacetylase activity, which is acquired through its interaction with HDAC1 [51]. This conclusion was supported by studies from another laboratory that found that HDAC1 protected neurons in cell culture and mouse models of Alzheimer disease and ischemic stroke [52]. Hence the idea of HDAC1 as a “molecular switch” between neuronal survival and death has been proposed [48], whereby HDAC1 can be either neuroprotective or neurotoxic depending on its interacting partner.

## 5. HDAC1 and HDAC3 in Polyglutamine Diseases

Polyglutamine expansion disorders are a group of nine inherited neurodegenerative diseases that are caused by mutations in CAG repeat tracts within coding regions of different disease genes. These include, Huntington’s disease (HD), dentatorubral-pallidoluysian atrophy, spinal and bulbar muscular atrophy (SBMA) and six of the spinocerebellar ataxias (SCAs), types 1, 2, 3, 6, 7 and 17. An increasing body of evidence suggests that epigenetic dysregulation is involved in the pathology of polyglutamine diseases. A common characteristic of these diseases is aberrant transcriptional regulation, due to the disrupted function of histone-modifying complexes and altered interactions of the polyglutamine-expanded disease proteins with chromatin-related factors. In particular, several studies have provided evidence that the chromatin acetylation status is greatly impaired in polyglutamine disorders, a common mechanism being the loss of function of a specific HAT: the CREB-binding protein (CBP). These features have been reviewed previously [1,2,3,53,54,55,56], and have provided the rationale for the proposed use of HDAC inhibitors as a relevant treatment approach. More recently, individual HDAC proteins, in particular HDAC1 and HDAC3, have been specifically linked to different polyglutamine disorders, as outlined below.

### 5.1. Huntington’s Disease

HD is an inherited, progressive neurodegenerative disorder characterized by chorea, movement dysfunction, cognitive impairment, and behavioral disturbances [57]. The prevalence rate in the US is approximately 5 per 100,000 people. The disease mutation in HD is within the CAG repeat region located in exon 1 of the *HTT* gene, resulting in an expanded polyglutamine tract near the N-terminus of the encoded huntingtin protein [57]. Disease onset is correlated with CAG repeat length (threshold ~39 repeats, with longer expansions resulting in earlier onsets) [58], although other disease modifiers are thought to affect age of onset, which is typically in 40s [59]. Typical HD is characterized by weight loss, cognitive disorders and motor impairment, including the hallmark feature of chorea (involuntary jerky movements of the face and limbs), and gait abnormalities. The disease lasts 15 years on average, until the death of the patient. Several lines of evidence have implicated HDAC1 and/or HDAC3 in the pathology of HD.

#### 5.1.1. Expression Levels of HDAC1 and HDAC3 in HD Tissues

Several studies have measured HDAC1 and HDAC3 mRNA and protein expression levels in different HD mouse models, with differing results depending on the animal model. An initial study examining the expression of a panel of HDAC enzymes showed increased levels of HDAC1 protein in cortical samples from R6/2 mice at both 4 and 12 weeks of age, and in the striatum of 9 week-old R6/2 mice [21]. This finding was validated by a separate group who also found elevated levels of HDAC1 protein in the striatum of R6/2 mice, but not in other tissues [48]. Similar changes in HDAC1 protein were not found in a full-length HD model, CAG140 knock-in mice [21]. A study by our group showed increased nuclear expression of HDAC1, but reduced cytosolic expression, in the cortex of 18-week old N171-82Q transgenic mice [31], whereby no changes in the related HDAC2 protein, nor HDAC4 or HDAC7, were found [31]. Elevated HDAC1 mRNA levels were detected by microarray analysis in the cortex, striatum and cerebellum of R6/2 mice [60,61], and via qPCR in the striatum of 18 week old N171-82Q transgenic mice (Jia H. and Thomas E.A., manuscript in preparation). No change in HDAC1 mRNA levels were reported in the cortex of 9 week old R6/2 transgenic mice [22], although later time-points were not tested.

HDAC1 expression has been measured in human brain samples, with inconsistent results [20,21,62]. HDAC1 mRNA was found to be elevated in microarray studies on human HD caudate and cortex [62]. In studies measuring protein expression, HDAC1 levels in human HD brain, were not found to be significantly different, as determined by immunohistochemistry; however, only two controls were used for the comparison the immunoreactivity signals were low [20]. Another study reported highly variable HDAC1 protein expression levels in HD human brain samples [21]. One possible reason could be that the data were not controlled for disease grade or age [21].

Expression levels of HDAC3 protein were not found to be significantly different in cortical samples from 4 and 12 week old R6/2 transgenic mice, although the striatum showed a non-significant increase at 9 weeks of [21]. With regards to protein localization, HDAC3 levels, similar to HDAC1, were found to be higher in nuclear fractions from in the cortex of 18-week old N171-82Q transgenic mice compared to wt littermates, whereas no significant differences in the expression of other HDACs, HDAC2, HDAC4 or HDAC7, were detected [31]. These selective increases in nuclear HDAC 1 and 3 expression, provide an interesting rationale for the efficacy of HDAC1/3-targeting inhibitors in HD models (see below). No changes in HDAC3 mRNA levels were reported in cortex of 9 week old mice [22], although later time-points were not tested in that study. However, elevated HDAC3 mRNA levels were detected in striatum of late-stage 18 week old N171-82Q transgenic mice (Jia H. and Thomas E.A., manuscript in preparation).

#### 5.1.2. HDAC3 Binds Huntingtin Protein

Recent studies have identified HDAC3 as an essential player in mutant huntingtin-induced neurodegeneration [63]. In this study, normal, wt huntingtin protein, but not the mutant form, was found to interact with HDAC3. Accordingly, expression of mutant huntingtin liberates HDAC3 from huntingtin, thus de-repressing its neurotoxic activity [63]. Further, mutant huntingtin neurotoxicity was inhibited by the knockdown of HDAC3 and markedly reduced in HDAC3-deficient neurons [63]. A reduction in huntingtin-HDAC3 interaction is also seen in neurons exposed to other apoptotic stimuli and in the striatum of R6/2 HD mice [63]. The robust interaction between huntingtin and HDAC3, along with the ability of mutant huntingtin to disrupt this interaction, provides an interesting explanation for both the loss-of-function and gain-of-toxic-function mechanisms proposed for HD.

#### 5.1.3. HDAC1 and Regulation of Huntingtin Clearance

One therapeutic approach in HD, as well as other protein aggregate disorders, is to improve the degradation of accumulated mutant proteins. Studies have implicated an important role for autophagy in the clearance pathway for mutant huntingtin fragments [64,65]. In particular, increased acetylation at lysine 444 of the huntingtin protein has been shown to facilitate trafficking of mutant huntingtin into autophagosomes, subsequently improving protein clearance and reversing the toxic effects of mutant huntingtin in primary striatal and cortical neurons, as well as in a transgenic *C*. *elegans* HD model [66]. This particular lysine residue was found to be regulated by the opposing activities of CBP and HDAC1. Specifically, overexpression of HDAC1 decreased acetylation of mutant huntingtin and knockdown of endogenous HDAC1 by shRNA significantly increased acetylation of mutant huntingtin at lysine 444 [66]. Increased acetylation of huntingtin was associated with improved clearance by the autophagosome. These findings were supported by a separate group of researchers who demonstrated that down-regulation of HDAC1 is essential for the degradation of mutant huntingtin through lithium-induced autophagic pathway [67].

#### 5.1.4. Genetic Knock-Down Studies in HD

The roles of individual HDACs in HD have been explored in genetic knock-down studies. Studies on *C*. *elegans* have demonstrated HDAC subtype-specific effects related to the huntingtin-mediated neuropathology. A report by Bates *et al.* [68] has shown that HDA-1 and HDA-3 (worm HDACs representing orthologues of HDAC1 and HDAC2, respectively) modulates polyglutamine toxicity in *C*. *elegans* neurons expressing a human huntingtin fragment with an expanded polyglutamine sequence [68]. These authors found that HDA-1 and HDA-3 had different targets with opposing effects on toxicity: HDA-1 showing neuroprotective effects and HDA-3, neurotoxic [68]. Genetic knock-down studies have also been carried out in a Drosophila model of HD that expresses mutant human huntingtin exon 1 protein (Httex1p Q93) in all neurons. Results showed that knock-down of *Rpd3* (Drosophila homolog of HDACs 1–3), but not *Hdac3* alone, was neuroprotective in Httex1p-induced fly eye neurodegeneration [69]. Among the NAD(+)-dependent class III deacetylases, genetic or pharmacological reduction of sirtuin 2 (*Sir2*) also provided neuroprotection to huntingtin-challenged flies, and that even greater neuroprotection was achieved when *Rpd3* and *Sir2* were simultaneously reduced [69]. Similar studies in mouse models have also been attempted, but with greatly difficulty, given that both *Hdac1* and *Hdac3* null mutants are embryonic lethal [28,39]. Genetic reduction of HDAC3 using *Hdac3 (+/−)* heterozygous mice has been reported, and was not found to ameliorate disease phenotypes when crossed with R6/2 transgenic mice; however the overall protein levels of HDAC3 were only reduced 20%, hence it is likely that the these reductions were not sufficient to observe significant changes in HD phenotypes [70]. Knock-down of *Hdac1* in a similar context has not been tested.

### 5.2. Spinocerebellar Ataxias

Six of the spinocerebellar ataxias (SCAs) are classified as polyglutamine disorders [71]; hence, these are caused by expanded CAG repeats in the coding regions of the relevant disease genes. All of these are autosomal dominant, progressive neurodegenerative diseases with predominantly adult-onset. Similar to HD, the CAG repeat threshold for toxicity is around 40, except for SCA3, where most of the clinically diagnosed patients show expansions of greater than 55 [72]. For at least three of the SCAs, the corresponding disease proteins have been found to interact specifically with HDAC3.

#### 5.2.1. Spinocerebellar Ataxia Type 1 (SCA1)

Spinocerebellar ataxia 1 (SCA1) is caused by glutamine repeat expansion in the ataxin 1 (*ATXN1*) gene [73]. SCA1 pathology is characterized by ataxia, progressive motor deterioration, and loss of Purkinje cells in the cerebellum [73]. Ataxin 1 has been implicated in transcriptional regulation. A genetic screen in *Drosophila* expressing full-length ataxin-1 revealed interactions between ataxin 1 and several transcriptional corepressors, including Sin3 and Rpd3 [74]. Further studies using co-immunoprecipitation showed that wt ataxin 1 interacts with components of the co-repressor NCoR/SMRT complex, and, specifically with the mammalian HDAC3, but not other HDAC proteins [75]. Ataxin 1 was also found to interact selectively with HDAC3 *in vivo*. In this case, however, the HDAC3-ataxin 1 interaction was not polyglutamine-dependent, which could suggest that inhibition of HDAC3 in this context may interfere with normal ataxin 1 regulatory properties.

#### 5.2.2. Spinocerebellar Ataxia type 3 (SCA3)

Spinocerebellar ataxia type 3 (SCA3), also known as Machado-Joseph disease, is caused by an unstable CAG repeat expansion in the ataxin-3 (*ATXN3*) gene leading to an expansion of polyglutamines in the C-terminus of the corresponding protein, ataxin-3 [72,76]. SCA3 is the most frequent subtype of autosomal dominant inherited spinocerebellar ataxias and is characterized by progressive gait and limb ataxia and ocular movement abnormalities. The disease protein ataxin-3 has been associated with the ubiquitin-proteasome system and transcriptional regulation. With regards to chromatin regulation, previous studies have shown that normal ataxin 3 binds to target DNA sequences in specific chromatin regions of the matrix metalloproteinase-2 gene promoter and represses transcription by recruitment of the HDAC3/NCoR complex. Further, it was shown that both normal and expanded ataxin 3 physiologically interact with HDAC3 and NCoR in a SCA3 cell model and in human pons tissue; however, normal ataxin 3-containing protein complexes showed increased histone deacetylase activity, whereas polyglutamine-expanded ataxin 3-containing complexes had reduced deacetylase activity in target chromatin regions [77]. The broadly-acting HDAC inhibitor, sodium butyrate, was found to reverse transcriptional downregulation and ameliorates ataxic symptoms in a transgenic mouse model of SCA3 [78]. The consequences of selective HDAC3 inhibition, however, are less clear, although it is possible that disruption of the HDAC3 repressor complex by polyglutamine-expanded ataxin 3 may liberate HDAC3 to elicit neurotoxic effects as observed in HD.

#### 5.2.3. Spinocerebellar Ataxia Type 7 (SCA7)

Spinocerebellar ataxia type 7 (SCA7) is caused by a toxic polyglutamine expansion in the N-terminus of the protein ataxin 7 (*ATXN7*) [71]. SCA7 is clinically characterized by progressive abnormalities of gait and limb movement due to neuronal loss within the cerebellum and brainstem. Ataxin 7 has a known function in the histone acetylase complex, Spt/Ada/Gcn5 acetylase (STAGA) chromatin-remodeling complex. One recent study coexpressed each HDAC family member in the presence of ataxin 7 and found that only HDAC3 increased the posttranslational modification of normal and expanded ataxin 7 [79]. Specifically, HDAC3 was found to physically interact with, and stabilize, ataxin 7, and the physical interaction of HDAC3 with normal and polyglutamine-expanded ataxin 7 affects the toxicity in a polyglutamine-dependent manner [79]. Further, Duncan and colleagues detected robust expression of HDAC3 in neurons and glia in the cerebellum of wt mice, and increased levels of HDAC3 protein in the cerebellum of SCA7 mice [79]. This study implicates HDAC3 and ataxin-7 interaction as a target for therapeutic intervention in SCA7, strongly suggesting that selective HDAC3 inhibitors could be useful for SCA7.

## 6. Selective HDAC Inhibitors

Over the past 5 years, there has been great progress in identifying isotype-selective HDAC inhibitors. Comprehensive reviews on the design and discovery of selective HDAC inhibitors have been covered elsewhere [80,81,82,83,84], hence, will only be mentioned briefly, herein.

Small molecule HDAC inhibitors which inhibit the zinc dependent classes of HDACs, fall into 4 main classes according to their chemical structure: hydroxamates, cyclic peptides, short chain fatty acids and benzamides. The hydroxamates and cyclic peptides are both thought to bind to the zinc ion in the catalytic domain of HDACs thus inactivating both class I and II HDACs [85]. A large majority of hydroxamate HDAC inhibitors act as pan-HDAC inhibitors, and do not show selectivity within the zinc dependent classes of HDAC enzymes [53]; however due to the large variation in chemical structure among peptides, peptide HDAC inhibitors can elicit inhibition of different HDAC isoforms, including HDACs 1–3 [80]. The short chain fatty acids are relatively small, simple structured compounds and most share similar HDAC isoform inhibition profiles: inhibiting the action of classes I and IIb HDACs [86]. The most notable drugs within this class are valproate (valproic acid), sodium butyrate and phenylbutyrate. Finally, the benzamides represent a new relatively selective class of HDAC inhibitors [84]. Examples of this class include both MS-275 and CI-994, which selectively inhibit HDAC1 (and HDAC3 to a lesser extent) over other HDAC isoforms [80,84]. In particular, members of the pimelic 2-aminobenzamide family inhibitors selectively target HDACs 1 and 3 and have been most widely studied for this potential benefit in Friedreich’s ataxia and HD (see below).

## 7. Selective HDAC Inhibitors in Polyglutamine Disorders

Over the past decade, numerous studies have identified HDAC inhibitors as candidate drugs for the treatment of polyglutamine disorders (reviewed in [3,53,87]). Initial preclinical studies testing HDAC inhibitors for therapeutic purposes in polyglutamine disorders were carried out using broad-spectrum HDAC inhibitors. Compounds such as SAHA, phenylbutyrate and sodium butyrate have been shown to impart beneficial effects in cell, *Drosophila*, and mouse models of polyglutamine disorders, including HD [88,89,90,91,92], SBMA [93] and SCA3 [79] and SCA7 [94]. However, the clinical use of these broadly-acting compounds for neurodegenerative disorders is limited by their known toxicity. Hence, it is becoming clear that inhibitors with subtype-selectivity may prove more beneficial for targeting neurological disease symptoms and minimizing harmful side effects.

### 7.1. Class I Specific HDAC Inhibitors

Several class I-specific HDAC inhibitors are commercially available, including the FDA-approved drug, valproic acid (VPA), which has a long and established history of efficacy in the treatment of bipolar disorder, but was later shown to have HDAC inhibitory properties, in particular, inhibition of class I HDACs [95,96]. VPA has been tested in different model systems for HD and SCA3, with positive results (Table 1). Two different studies have shown that VPA ameliorates disease phenotypes in N171-82Q transgenic mice and YAC128 mice [97,98], with the latter study showing potentiating effects in the presence of the mood stabilizer lithium [98]. VPA was also tested in both Drosophila and cell SCA3 models [99]. VPA was found to ameliorate eye depigmentation, alleviate climbing disability, and extend the average lifespan of SCA3 transgenic Drosophila expressing a truncated form of ataxin-3 containing a 78-residue expanded polyglutamine tract (MJDtr-Q78) [99]. Further, VPA reduced the early apoptotic rate in MJDtr-Q68- expressing cells in association with increased the acetylation levels of histone H3 and histone H4 [99].

Early clinical trials in humans, dating back to 1977, have examined VPA for its potential gamma-aminobutyric acid (GABA) elevating properties, although the results did not show significant clinical benefit to HD patients [100,101]. More recent studies, however, have shown that some benefit can be accrued in HD patients either as specific monotherapy [102] or as a combination therapy with olanzipine [103].

Another class I HDAC inhibitor was shown to have beneficial effects in two different HD mouse models. d-β-Hydroxybutyrate (DβHB), an endogenous HDAC inhibitor of class I HDACs, showed neuroprotective effects in the 3-nitropropionic acid (3-NP) toxic mouse model of HD and in R6/2 transgenic mice, ameliorating histone hypoacetylation and improving locomotor activity and premature death [104].

### 7.2. HDAC1/HDAC3-Targeting Inhibitors

Subtype-selective, benzamide-type, HDAC inhibitors have been developed as a therapeutic approach for Friedreich’s ataxia [105,106]. Several compounds in this class have been shown to selectively and potently inhibit HDAC1 and HDAC3 [31,106,107]. These selective inhibitors have also been tested in HD model systems (Table 1). Much work has focused on one compound, HDACi 4b, which preferentially targets HDAC1 and HDAC3 enzymes and exhibits low *in vitro* and *in vivo* toxicity [31,60]. Initial studies demonstrated that HDACi 4b could ameliorate body weight loss, motor dysfunction and striatal volume decline in R6/2 transgenic mice, when administered in drinking water [60], with later studies also showing improvement in striatal volume loss and clasping behavior, via a similar drinking water paradigm [108], although this delivery paradigm is not optimal [108,109]. Improved results were found when HDACi 4b was administered by s.c. injection, where drug treatment 2–3 times per week improved motor deficits and cognitive decline in N171-82Q transgenic mice [110]. HDACi 4b, along with a related inhibitor, HDACi 874, were both found to prevent mutant huntingtin aggregation in several brain regions [110]. HDACi 4b was also tested in cell and fly HD models systems. Drug treatment improved the metabolic deficit exhibited by immortalized, striatal, ST*Hdh*^Q111^ cells and ameliorated eye neurodegeneration in *Drosophila* expressing N-terminal human huntingtin fragments (Httex1p Q93) [31].

Other HDAC1/HDAC3-targeting compounds have also been tested in HD model systems. These compounds showed beneficial effects in reducing eye neurodegeneration in HD *Drosophila*, improving metabolic function in ST*Hdh*^Q111^ striatal cells and reversing transcriptional deficits in the brains of R6/2 transgenic mice (31) (Table 1). Jia and colleagues also demonstrated in that study that HDAC3-selective compounds were effective in HD models. One compound RGFP136 prevented eye neurodegeneration in HD *Drosophila*, increased metabolic function in ST*Hdh*^Q111^ striatal cells and reversed transcriptional deficits of a subset of HD-related genes in R6/2 transgenic mice [31] (Table 1). Interestingly, RGFP136 was also found to significantly enhance persistent long-term memory in normal rats [111], suggesting improvement in cognitive function under normal conditions. Recently, another HDAC3-selective inhibitor, RGFP966, was tested in the N171-82Q transgenic mouse model, where it significantly prevented body weight loss, improved several parameters of motor function and ameliorated Huntingtin-elicited cognitive decline in N171-82Q transgenic mice [112]. These findings demonstrate that class I HDAC inhibitors are effective in suppressing pathogenic symptoms in various polyglutamine models with HDAC3-selective compounds exhibiting some of the strongest effects.

**Table 1:** Class I specific and isotype-selective HDAC inhibitors tested in HD model systems.

Class I-specific	HD Model and dose	Dose paradigm	Effects	Ref
Valproic Acid	N171-82Q transgenic mice	100 mg/kg; i.p.	Ameliorated premature death and locomotor activity deficits.	[97]
	N171-82Q transgenic mice	25 g/kg in diet	Ameliorated premature death and depressive-like behavior.	[98]
	YAC128 transgenic mice	25 g/kg in diet	Ameliorated body weight gain and anxiety-like behavior.	[98]
	Drosophila MJDtr-Q78	0.5–2 mM in diet	Prevented eye depigmentation, alleviated climbing disability, and extended the lifespan.	[99]
	MJDtr-Q68- expressing cells	0.5–2 mM in culture	Reduced apoptosis.	[99]
d-β-HB (d-β-H-butyrate)	3-NP mouse model	1.6 mmol/kg/day; minipump	Improved spontaneous locomotor activity.	[104]
	R6/2 transgenic mice	1.6 mmol/kg/day; minipump	Ameliorated premature death.	[104]
**HDAC1/HDAC3**	**HD Model**	**Dose paradigm**	**Effects**	**Ref**
HDACi 4b	R6/2 transgenic mice	150 mg/kg/day; drinking water	Ameliorated body weight loss and locomotor deficits.	[60]
	R6/2 transgenic mice	150 mg/kg/day; s.c.	Prevented downregulation of a subset of HD-related genes.	[60]
	Drosophila Httex1p Q93	1–10 μM in diet	Ameliorated eye neurodegeneration.	[31]
	STHdhQ111 cells	0.3–10 μM in culture	Improved metabolic deficit.	[31]
	N171-82Q transgenic mice	50–100 mg/kg; s.c	Ameliorated body weight loss, locomotor deficits and cognitive decline.	[110]
	R6/2 transgenic mice	0.85 mg/ml; drinking water	Ameliorated striatal atrophy and clasping phenotype.	[108]
	N171-82Q transgenic mice	0.85 mg/ml; drinking water	No change in disease phenotypes.	[108]
HDACi 874	N171-82Q transgenic mice	50 mg/kg; s.c.	Prevented mutant Htt aggregation.	[110]
HDACis 874, 968 and 974	Drosophila Httex1p Q93	1–10 μM in diet	Ameliorated eye neurodegeneration.	[31]
	STHdhQ111 cells	0.3–10 μM in culture	Improved metabolic deficit.	[31]
**HDAC3-selective**	**HD Model**	**Dose paradigm**	**Effects**	**Ref**
RGFP136	R6/2 transgenic mice	150 mg/kg; s.c.	Prevented downregulation of a subset of HD-related genes.	[31]
	Drosophila Httex1p Q93	1–10 μM in diet	Ameliorated eye neurodegeneration.	[31]
	STHdhQ111 cells	0.3–10 μM in culture	Improved metabolic deficit.	[31]
RFGP966	N171-82Q transgenic mice	50 mg/kg; s.c.	Ameliorated body weight loss, locomotor deficits and cognitive decline.	[112]
	Drosophila Httex1p Q93	1–10 μM in diet	Ameliorated eye neurodegeneration.	[31]
	STHdhQ111 cells	0.3–10 μM in culture	Improved metabolic deficit.	[31]
**HDAC1-selective**	**HD Model**	**Dose paradigm**	**Effects**	**Ref**
228	R6/2 transgenic mice	150 mg/kg; s.c.	Prevented downregulation of a subset of HD-related genes.	[31]
233, 941 and MS-275	Drosophila Httex1p Q93	1–10 μM in diet	Ameliorated eye neurodegeneration.	[31]
	STHdhQ111 cells	0.3–10 μM in culture	Improved metabolic deficit.	[31]

## 8. Mechanisms of Action of HDAC Inhibitors

There are several mechanisms by which HDAC inhibitors can be acting to improve disease phenotypes. In the most simplistic view, it is expected that inhibiting HDAC enzymes will increase histone acetylation and subsequent gene expression. This could be in the context of reversing a disease-induced histone hypoacetylation, as has been implicated in polyglutamine disorders, resulting in transcriptional reactivation of silent genes, or increase the expression of disease-modifying or neuroprotective genes. However, several microarray studies have demonstrated that HDAC inhibitors can cause both up- and down-regulation of gene expression patterns, with little predictive value [60,92,113]. These results suggest that it is likely that HDAC inhibition may alter the expression of other regulatory enzymes and/or co-factors, which subsequently act as activators or repressors of gene activity. Accumulating evidence suggests that many HDACs, including class I HDACs, can also deacetylate non-histone proteins [114], which may contribute to their beneficial properties. These are discussed below.

### 8.1. Chromatin/Transcription-Related Mechanisms of HDAC1/3 Inhibitors

One primary consequence of HDAC inhibition is the elevation of histone acetylation, thereby promoting an open chromatin structure and facilitation of gene transcription. Accordingly, studies have demonstrated that different types of HDAC inhibitors elevate histone acetylation on both local and/or global level [21,60,105,106], and microarray studies have demonstrated that HDAC inhibitors do indeed elicit effects on gene expression. However, contrary to early predictions, HDAC inhibitors do not cause global upregulation of gene transcription. Rather, previous expression profiling studies have demonstrated a relatively small number of genes (2%–10%) modulated by HDAC inhibitors [113]. This phenomenon has been demonstrated in several studies that have shown beneficial effects of HDAC inhibitors in disease models [60,91]. Microarray studies have revealed potential mechanistic information regarding the types of genes and pathways that are altered by drug treatment. Implications for the ubiquitination- and neuroprotective-related gene expression changes are described further below.

#### 8.1.1. Ubiquitination-Related Gene Expression

**Figure 2:**
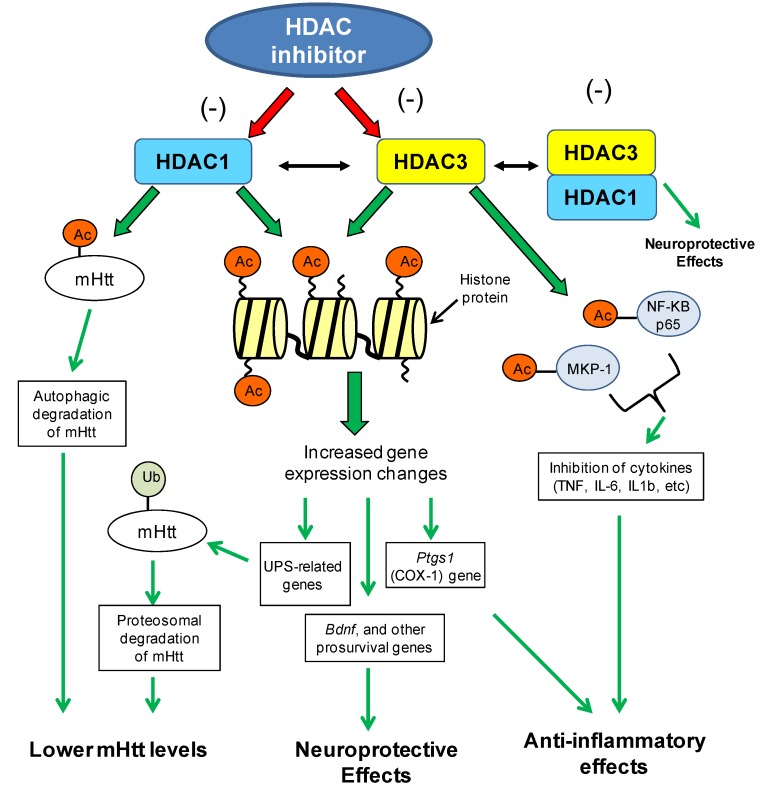
Potential chromatin- and non-chromatin-related mechanisms of HDAC1/HDAC3-targeting inhibitors in the context of Huntington’s disease. Different mechanisms associated with inhibition of HDAC1 and HDAC3 enzymes can lead to lowered mutant huntingtin (mHtt) levels, neuroprotective effects or anti-inflammatory effects, all of which can contribute to the improved disease phenotypes observed in HD model systems (see text for details). Neuroprotection by HDAC inhibitors could also be mediated through the inhibition of the HDAC1–HDAC3 interaction and subsequent toxicity of this interaction (see Ref 63). Acetyl group (Ac); Mutant huntingtin (mHtt); ubiquitin proteasomal system (UPS); brain-derived neurotrophic factor (Bdnf); prostaglandin-endoperoxide synthase 1 (Ptgs1/COX1); mitogen-activated protein kinase phosphatase-1 (MKP-1); nuclear factor kappa-light-chain-enhancer of activated B cells (NF-KB).

Functional and network analysis of microarray data from HDACi 4b-treated R6/2 transgenic mice implicated ubiquitination as significantly associated with HDAC inhibitor treatment [110]. Several genes related to the ubiquitin-proteasome pathway were found to be regulated by 4b at different time points of treatment and in different brain regions. In the cortex, 4b normalized Htt-induced deficits in the expression of Ubiquitin-conjugating enzyme E2K (*Ube2k*, a.k.a. Hip2), Ubiquitin-like modifier activating enzyme 7 (*Uba7)* and Ubiquitin specific peptidase 28 (*Usp28*), at 3 days and 6 weeks of treatment but not at the late-stages of disease. In the striatum, 4b caused significant increases in the expression of Ubiquilin 2 (*Ubqln2*) and *Usp28* in N171-82Q mice after 12 weeks of treatment. These genes play vital roles in the ubiquitin-proteasome system. The ubiquitin-proteasome system is the main intracellular pathway for regulated protein turnover. This system is essential for maintaining cellular homeostasis and in regulating fundamental cellular events, such as cell division, apoptosis and neuronal functioning [115,116]. It is thought that dysregulated ubiquitination of target proteins could play a role in neurodegenerative disorders, many of which are pathologically characterized by the presence of ubiquitin-positive protein aggregates, suggesting impairment of the ubiquitin-proteasome system [115,117,118,119,120]. In the Jia *et al.* study, the drug-elicited changes in the expression of ubiquitin genes were correlated with reduced mutant Htt aggregate formation in the brains of N171-82Q transgenic mice [110]. Increased ubiquitination of Htt protein has been linked to reduced toxicity of mutant Htt protein, most likely through increased clearance by the proteasome [121,122,123]. These findings implicate HDAC1 and HDAC3 in chromatin-mediated repression of ubiquitination related genes, whereby inhibition of these subtypes promotes upregulation of the ubiquitin proteasomal system (see Figure 2).

Pathological inclusions containing misfolded proteins are a prominent feature common to many age-related neurodegenerative diseases besides HD, including Parkinson’s disease, Alzheimer’s disease and amyotrophic lateral sclerosis. Hence, the ability of HDAC1/3 inhibitors to promoter/increase the ubiquitin-proteasomal system could have clinical relevance for several neurodegenerative disorders associated with protein aggregation.

#### 8.1.2. Brain-Derived Neurotrophic Factor Gene (BDNF) Expression Changes

Several studies have demonstrated that HDAC inhibitors could be neuroprotective agents, hence providing an explanation for the use of these compounds in a wide range of neurological disorders (reviewed in [124]). A proposed mechanism is evidenced to be via regulation of neuronal or glial gene expression, most notably, the expression of brain-derived neurotrophic factor (BDNF) (Figure 2). VPA in particular has been shown to promote neurogenesis, neurite outgrowth, synaptic plasticity and neuroprotection, with evidence supporting a role of elevated BDNF in these effects [125,126,127,128]. Initial studies found that VPA upregulates the expression of BDNF, as well as glial cell line-derived neurotrophic factor (GDNF) from astrocytes in association with neurotrophic effects on dopamine neurons [127]. Similar to VPA, broadly-acting HDAC inhibitors were also found to increase GDNF and BDNF transcripts in astrocytes, in association with elevated *Bdnf* promoter-associated histone H3 acetylation [125].

VPA-elicited elevation of BDNF expression has also been demonstrated in neurons. Microarray analysis on rat cortical neurons found that VPA-treatment markedly increased *Bdnf* gene expression, which was validated by qPCR analysis and assays showing increased acetylation of histones H3 and H4 at *Bdnf* promoters I and IV [129]. In a separate study on cortical neurons, VPA treatment elicited activation of *Bdnf* promoter IV [130], implicating HDAC1 in the effect. In that study, *Bdnf* promoter IV activity was stimulated after transfection with an HDAC1-specific siRNA [130] and in HDAC1 siRNA-treated neurons, VPA failed to further enhance *BDNF* promoter IV activity. This is consistent with data that the expression of BDNF exon I–IX mRNAs is regulated by the NRSF/REST complex, an HDAC1-containing co-repressor complex [131]. Additional studies reported that VPA upregulated BDNF in neural stem cells from *Npc1*(−/−) mice [132] and in retinal ganglion cells [128].

### 8.2. Non-Chromatin Mechanisms Associated with HDAC1 and HDAC3

The increasing number of acetylated non-histone proteins suggests an important role for HDACs in the regulation of cellular processes beyond chromatin and gene expression [113]. In particular, both HDAC1 and HDAC3 have been shown to deacetylate non-histone proteins, notably transcription factors. One of the first non-histone targets identified for HDACs was p53, which has been widely linked to cancer and more recently, neurodegenerative disorders [133]. HDAC1 was found to bind p53 [134] and subsequently to deacetylate p53 both *in vitro* and *in vivo* [135,136]. Since then, the acetylation status of several transcriptions factors has been shown to be modulated by HDAC1, including YY1, Signal transducer and activator of transcription 3 (Stat3), androgen receptor, Myogenic differentiation 1 (Myod1), E2F transcription factor 1 (E2F1) and Smad family member 7 (Smad7) [113]. One particularly relevant non-histone target of HDAC1 for polyglutamine disorders is the huntingtin protein. As mentioned above, HDAC1 is responsible for the deacetylation of the huntingtin protein at lysine 444 [66]. That study by Jeong and colleagues demonstrated that specific knockdown of endogenous HDAC1 by shRNA, or treating with an HDAC inhibitor, increased acetylation of mutant huntingtin resulting in enhanced clearance [66] (Figure 2). Another acetylation site on huntingtin protein is the triad AcK9/pS13/pS16, which has also been linked to autophagic degradation [137] and can be regulated by HDACi 4b treatment [110].

Several studies have demonstrated a role for HDAC3 in inflammatory signaling. This is pertinent in light of emerging evidence implicating immune activation and inflammation as playing important roles in the pathogenic mechanism of neurodegenerative disorders. Specifically, HDAC inhibitors targeting class I (VPA or MS-275) or HDAC3 (MI192) have been shown to decrease the expression of many cytokines, including TNF, IL-6 and IL1b [138,139,140]. The mechanisms associated with these effects are likely to be complex, possibly involving both chromatin-association and non-chromatin associated effects (see Figure 2). One study demonstrated that *Hdac3*(−/−) cells display greatly increased constitutive expression of *Ptgs1* (COX-1), which encodes a key enzyme in the synthesis of prostaglandins and reactive electrophilic oxoderivatives (EFOXs), which show strong anti-inflammatory effects [141]. Other proposed mechanisms for HDAC3 include deacetylation of transcription factors or other non-histone proteins. Non-histone transcription factor targets for HDAC3 include Smad7, Myocyte enhancer factor 2 (MEF2), Mitogen-activated protein kinase phosphatase-1 (MKP-1), nuclear factor-kappaB (NFkB) [113,142,143,144], with two of these, MKP-1 and NFkB being implicated in inflammation.

MKP-1 is an essential, endogenous, negative regulator of innate immune responses and is regulated by post-translational protein acetylation. Recent studies have demonstrated that HDAC isoforms 1, 2 and 3 could regulate innate immune responses by deacetylating MKP-1 [143]. Further, pharmacologic inhibition of HDACs 1, 2, and 3 with the HDAC inhibitor, MS-275, was found to decrease LPS-induced expression of TNF-α, IL-1β, iNOS, and nitrite synthesis in macrophage cells [144].

Another important non-histone target for HDAC3 is nuclear factor-kappaB (NFκB), which is widely known to play important roles in inflammation and immune responses in the CNS [145]. The activity of NF-κB (a heterodimer of RelA[p65] and p50 proteins) is controlled by acetylation, which regulates its interaction with IκBα and subsequent activity [143]. Studies in HEK293 cells using RNAi- knockdown and gene overexpression, have indicated a role for HDAC3 in the expression of inflammatory genes, in which it was found that HDAC3 is a global regulator of the transcriptional IL-1 response, which is linked to NFκB activity. The stimulatory function of HDAC3 in inflammatory gene expression was found to involve a specific interaction with, and deacetylation of, NFκB p65 at several lysines residues [146]. This effect was notably independent from the gene regulatory effects mediated by the broad-spectrum HDAC inhibitor trichostatin A [146], hence implicating a non-chromatin effect. These data describe a novel function for HDAC3 as a co-activator in inflammatory signaling and might help to explain the anti-inflammatory effects frequently observed for HDAC inhibitors in clinical use.

## 9. Conclusions

Despite being part of large family of enzymes, individual HDAC proteins clearly have distinct biological roles in cellular function, and in the pathological involvement of polyglutamine disorders. This review has summarized the involvement of HDAC1 and HDAC3 in neurodegenerative processes and transcriptional dysregulation associated with different polyglutamine diseases, which has provided a basis for the use of selective HDAC inhibitors targeting these subtypes for therapeutic purposes. While our results indicate that pharmacological targeting HDAC1 and HDAC3, would be most beneficial for HD therapeutics, this does not preclude the involvement of other HDAC enzymes, such as the sirtuins (class III HDACs), in the pathology of, or therapeutic application for, other polyglutamine disorders.

While the consequences of HDAC inhibition may act to correct pertubations in histone acetylation homeostasis, restore transcriptional balance to disease or disease-modifying genes, or activate neuroprotective mechanisms, additional functional studies are needed in order to fully understand the mechanisms associated with the beneficial effects of selective HDAC inhibitors, including the use of “acetylome” analyses to identify specific substrates and to further define the pathways in which specific HDAC enzymes are involved.
